# Shale pore characteristics and their impact on the gas-bearing properties of the Longmaxi Formation in the Luzhou area

**DOI:** 10.1038/s41598-024-66759-7

**Published:** 2024-07-23

**Authors:** Jing Li, Hu Li, Wei Jiang, Molun Cai, Jia He, Qiang Wang, Dingyuan Li

**Affiliations:** 1 Institute of Geological Exploration and Development of CNPC Chuanqing Drilling Engineering Company Limited, Chengdu, 610051 China; 2 School of Economics, Sichuan University of Science and Engineering, Yibin, 644000 China; 3 National Key Laboratory of Oil and Gas Reservoir Geology and Exploitation (Southwest Petroleum University), Chengdu, 610500 China; 4 Shale Gas Exploration and Development Department of CNPC Chuanqing Drilling Engineering Company Limited, Chengdu, 610051 China; 5 Shale Gas Research Institute, PetroChina Southwest Oil and Gasfield Company, Chengdu, 610051 China

**Keywords:** Shale gas, Pore structure, Gas-bearing properties, Thermal evolution, Mineral composition, Geochemistry, Solid Earth sciences

## Abstract

Deep shale has the characteristics of large burial depth, rapid changes in reservoir properties, complex pore types and structures, and unstable production. The whole-rock X-ray diffraction (XRD) analysis, reservoir physical property parameter testing, scanning electron microscopy (SEM) analysis, high-pressure mercury intrusion testing, CO_2_ adsorption experimentation, and low-temperature nitrogen adsorption–desorption testing were performed to study the pore structure characteristics of marine shale reservoirs in the southern Sichuan Basin. The results show that the deep shale of the Wufeng Formation Longyi_1_ sub-member in the Luzhou area is superior to that of the Weiyuan area in terms of factors controlling shale gas enrichment, such as organic matter abundance, physical properties, gas-bearing properties, and shale reservoir thickness. SEM is utilized to identify six types of pores (mainly organic matter pores). The porosities of the pyrobitumen pores reach 21.04–31.65%, while the porosities of the solid kerogen pores, siliceous mineral dissolution pores, and carbonate dissolution pores are low at 0.48–1.80%. The pores of shale reservoirs are mainly micropores and mesopores, with a small amount of macropores. The total pore volume ranges from 22.0 to 36.40 μL/g, with an average of 27.46 μL/g, the total pore specific surface area ranges from 34.27 to 50.39 m^2^/g, with an average of 41.12 m^2^/g. The pore volume and specific surface area of deep shale gas are positively correlated with TOC content, siliceous minerals, and clay minerals. The key period for shale gas enrichment, which matches the evolution process of shale hydrocarbon generation, reservoir capacity, and direct and indirect cap rocks, is from the Middle to Late Triassic to the present. Areas with late structural uplift, small uplift amplitude, and high formation pressure coefficient characteristics favor preserving shale gas with high gas content and production levels.

## Introduction

**Figure 1:**
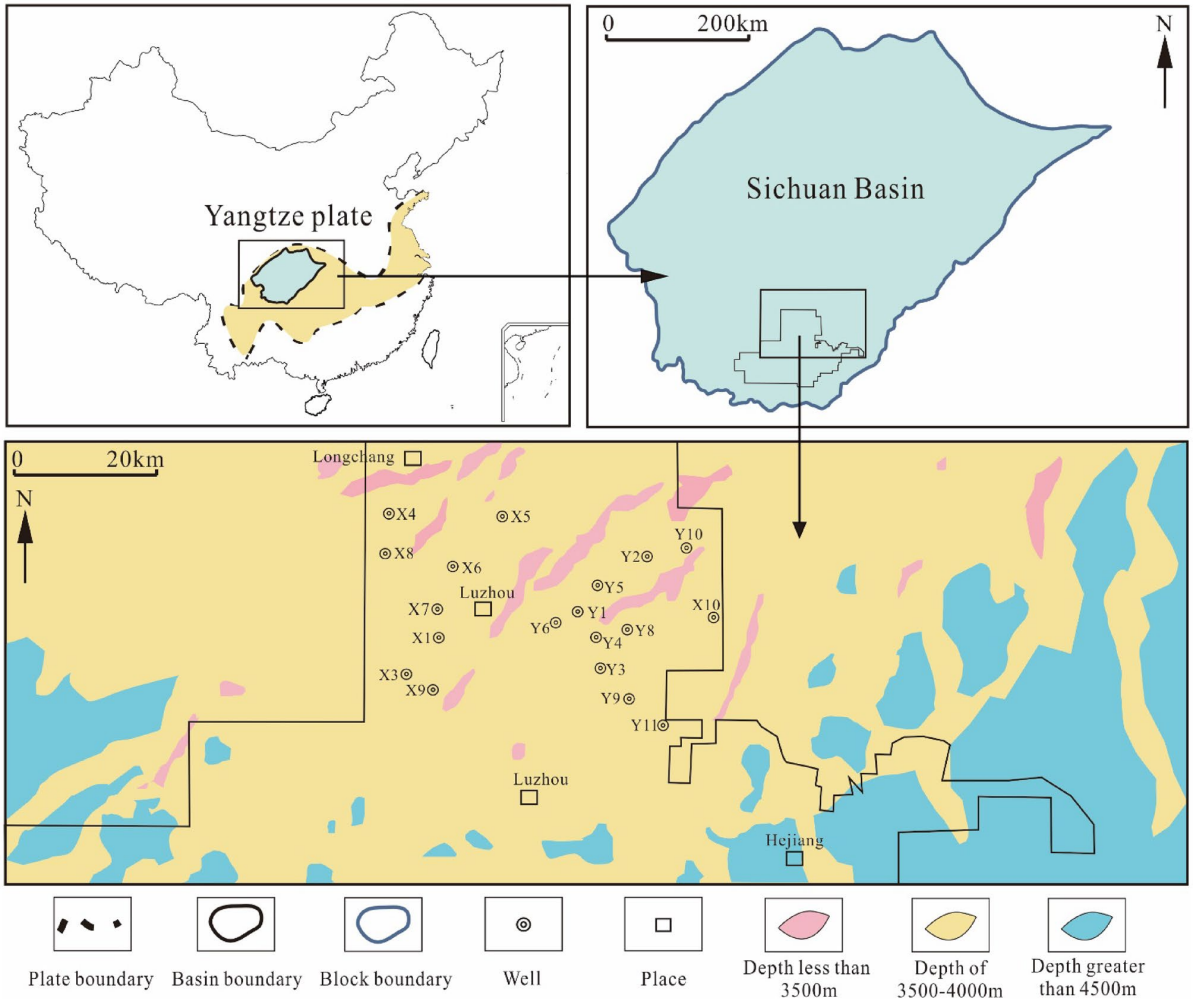
Location of the study area and the distribution of the wells (this figure is generated in CorelDRAW 2020 software, https://www.coreldrawchina.com/).

The bottom boundaries of the deep shale gas reservoirs in the Longmaxi Formation in the southern Sichuan area are generally buried at depths of 3500–5000 m. In recent years, PetroChina has increased the exploration and development of deep shale gas, achieving a series of exploration achievements^1,2^. For example, Well X2 in the Luzhou anticline structural belt obtained 137.9 × 10^4^ m^3^/day of gas during testing. However, various problems in actual exploration, development, and production remain^1,3,4^. While several high-yield wells have been discovered in deep shale gas areas, there have been low-yield wells with low gas production and estimated ultimate recovery (EUR) levels. The burial depths of Well Y3, Well Y8, and Well Y11 in the Luzhou deep shale gas area are larger than 4000 m (Fig. 1), with low test production, late gas breakthrough, and low production values at the same flowback rate. In ultradeep Well X14 (vertical depth 4900 m), when the burial depth is greater than 4500 m, the porosity of the reservoir greatly decreases, the pore characteristics and structure change significantly, and the gas content decreases. For physical properties between deep shale gas reservoirs and medium and shallow reservoirs in southern Sichuan, the porosity, pore type, and pore structure show the biggest differences, key factors determining the qualities of shale gas reservoirs^5,6^.

Shale reservoirs are important for shale gas generation and storage, and the pore structure has significant controlling effects on the gas occurrence state, seepage mechanism, desorption, and diffusion. Quantitative characterization of pore structure characteristics is a key issue in evaluating unconventional shale reservoirs and enhancing shale gas recovery^7,8^. With the rapid progress of shale gas exploration and development, many scholars have used scanning electron microscopy (SEM), nuclear magnetic resonance (NMR), high-pressure mercury intrusion, and gas adsorption methods to study shale reservoirs' microscopic pore structure characteristics. Combining Image J image processing technology and SEM with argon ion beam milling provides accurate and rapid research methods for quantitatively characterizing structural parameters such as pore morphology, pore size distribution, probability entropy, and pore area contribution^9–16^. Through the multiscale and full-scale quantitative characterization of reservoir pore structure characteristics and the quantitative evaluation of heterogeneity in the evolution of microscopic reservoirs, the enrichment of unconventional reservoirs, the state of oil and gas storage, and the accumulation law are analyzed and studied, achieving the efficient exploration and development of deep shale gas reservoirs.

Given the large burial depth of deep shale, rapidly changing reservoir storage capacity and connectivity, high pore structure complexity and irregularity, and unstable production, various studies and experiments are performed on the microscopic pore structure characteristics of shale gas reservoirs. The reservoir pore structure characteristics undergo multiscale quantitative characterization. The evolution characteristics of shale gas-controlling conditions are simulated by combining the stratigraphic burial and thermal evolution characteristics of hydrocarbon generation in the Luzhou area^17–19^. Lastly, the impacts of 3D effective sealing on the gas-bearing properties of shale gas are evaluated.

## Geological setting

**Figure 2:**
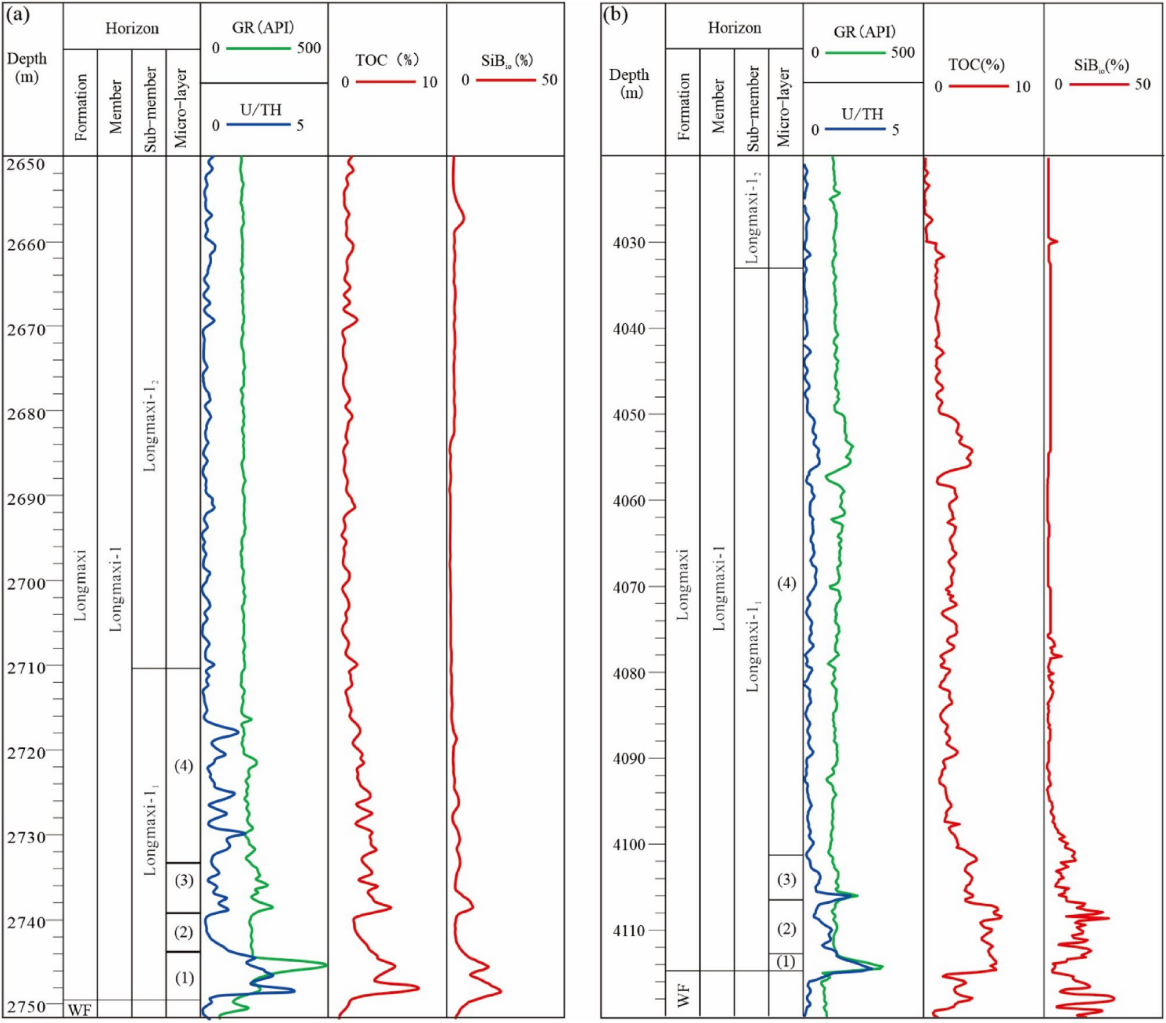
Stratigraphic histogram of the first Member of the Wufeng Formation–Longmaxi Formation. (**a**) Well B1, (**b**) Well X1 (this figure is generated in CorelDRAW 2020 software, https://www.coreldrawchina.com/).

The main exploration and development area is the shale reservoir in the Wufeng Formation–Longmaxi Formation in the southern part of the Sichuan Basin. Well B1 in the Weiyuan area is a typical well of middle-shallow shale gas in southern Sichuan, and Well X1 in the Luzhou area is a typical well of deep shale gas in southern Sichuan. As shown in Fig. 2, the lithological characteristics of the Upper Ordovician Wufeng Formation and the first Member of the Lower Silurian Longmaxi Formation are characterized by biotite-bearing grey carbonaceous shale below the interface with well-developed fauna fossils, including many brachiopods and echinoderm fossils, by organic-rich siliceous shale above the interface with abundant graptolite fossils, and by many siliceous radiolarians and siliceous sponge spicules^20,21^. Regarding the lithostratigraphic characteristics of the 1st and 2nd members of the Longmaxi Formation, the first Member of the Longmaxi Formation is mainly composed of gray-black calcareous shale and black shale intercalated with pyrite and calcareous strips, featuring underdeveloped shale bedding in the upper and middle parts and developed shale bedding at the bottom^22^. The second Member is mainly composed of gray calcareous shale.

## Samples and methods

### Samples

In this study, the cores were sampled from the Wufeng-Longmaxi Formation from four wells, namely, wells X1, X2, Y1, and Y2 in the Luzhou area and wells A1, A2, B1, and B2 in the Weiyuan area and were experimentally tested for the total organic carbon (TOC) content, degree of thermal evolution (Ro), whole-rock X-ray diffraction, shale porosity, pore type, microscopic pore structure, CO_2_ adsorption experiment, Nitrogen adsorption–desorption test and the high-pressure mercury porosimetry (Table 1). In addition, the drilling and logging data of these wells are from Petro China Southwest Oil & Gasfield Company.

**Table 1:** Test items and samples.

Test item	Test instrument	Samples number	
		Luzhou	Weiyuan
X-ray fluorescence	CIT-3000SY	310	248
TOC	CS230SH	22	20
Ro	AxioScope.A1 polarizing microscope + MSP.400 microspectrophotometer	20	15
Whole rock "X" diffraction	X’Pert Pro	25	23
Micro-pore structure	Helios 650	18	15
Porosity	CMS-300	16	12
CO_2_ adsorption experiment	Autosorb iQ Station 1	12	–
Nitrogen adsorption–desorption test	Micromeritics 3 Flex	12	–
The high-pressure mercury porosimetry	Autopore IV 9600	15	–

### Methods

In the CO_2_ adsorption experiment, CO_2_ gas at 0 °C (ice-water bath) is used, and according to the low-pressure isotherm adsorption curve, the micropores can be described in detail by the density functional theory (DFT) model. In this study, the Autosorb iQ Station 1 analyzer is used. The pore size range is 0.35–2 nm, the minimum detected specific surface area is 0.01 m^2^/g, and the pore volume is 0.1 uL/g. Before conducting gas adsorption testing to remove residual adsorbed water and volatile substances from shale samples, all samples were dried at 105 ℃ for 8 h. Then, an appropriate amount of samples were weighed and vacuum degassed at 105 ℃ for more than 12 h in a degassing station, followed by a 4-h vacuum degassing process. The sample particle sizes are determined by 40–60 mesh, and the test temperature is 273.15 K. The experimental test range is generally less than 2 nm, which is used to analyze the characteristics of shale micropores. The experiment is conducted according to the *Pore size* distribution and porosity of solid materials by mercury porosimetry and gas adsorption-Part 3: Analysis of micropores by gas adsorption (GB/T 21650.3-2011)^23,24^.

The low-temperature nitrogen adsorption–desorption test involves a Micromeritics 3 Flex analyzer. The experimental conditions are as follows: temperature of −195.8 ℃, pressure of 97.3–127.0 kPa, powder size of 0.28–0.18 mm, pore size range of 0.35–400 nm, and optimal pore size range of 1.7–50 nm. The pore type is determined according to the shape of the isotherm adsorption–desorption curve. Different pore size models have different calculation principles, and the mesopores and micropores can be described comprehensively by the Brunauer–Emmett–Teller (BET) equation and the Barrett–Joyner–Halenda (BJH) equation, respectively^25^.

The high-pressure mercury porosimetry involves an Autopore V 9600 high-pressure mercury intrusion porosimeter produced by Micromeritics. The test pressure range is 0.2–60,000 psi, the test pore throat range is 30 nm–1000 µm, and the sample is a 1 cm^3^ cube. Before the experiment, the samples are dried in an incubator for 72 h at a temperature of 80 °C. This experiment mainly determines shale samples' pore structure parameters and seepage characteristics. The pore diameter corresponding to each pressure point and the mercury injection amount are obtained through the Washburn equation, and the macropore distribution characteristics are obtained.

The SEM analysis with argon ion beam milling involves a LEICA EM RES102 argon ion beam milling system and a high-resolution field emission scanning electron microscope (FESEM) produced by FEI. The electron beam acceleration voltage is 20 V–30 kV, and the ion acceleration voltage is 500 V–30 kV. Focused ion beam scanning electron microscopy (FIB-SEM) is a FESEM with a gallium ion beam at 52° to the electron beam. The ion beam is placed perpendicular to the sample surface to cut the sample, and the electron beam is scanned at an angle of 38° to image the sample surface. The sample area is approximately 600 µm × 400 µm, which can accurately analyze the micropores of shale in the micro–nano range and distinguish the 1–2 nm nanopores. The experiment is according to the Analysis method of the petroleum and gas reservoir sandstone sample by scanning electron microscopy (GB/T 18295-2001) and the analytical method of a rock sample by scanning electron microscopy (SYT5162-2014).

Image J software was selected as the analysis software, which has the characteristics of simplicity, efficiency, and repeatability compared to traditional manual measurement methods in practical applications. The processed images can objectively represent the morphology of various pores and cracks in the images and quantitatively characterize pore structure parameters such as connectivity and geometric parameters.

## Results

### Organic carbon content, mineral composition, and porosity

The analysis (Table 2) shows that the TOC content of the samples in the study area is between 0.35 and 1.97%, with an average value of 1.12%. The porosity is between 0.57 and 6.34%, with an average value of 3.66%. Overall, the quartz content is the highest, followed by clay minerals and calcite, with an average quartz content of 51.3%, an average clay mineral content of 30.6%, and an average calcite content of 10.0%. Core analysis shows that the average clay content of the Longmaxi Formation is 35.8%; the clay mineral are mainly illite, followed by illite/smectite and chlorite, with a small amount of kaolinite. The contents of illite, illite/smectite mixed layer, and chlorite are relatively high, accounting for 15.56%, 6.44%, and 5.56% of the clay minerals and 6.43%, 3.08% and 2.47% of the total rock, respectively.

**Table 2:** Statistics of the organic carbon content, porosity, and mineral composition in the study area.

Area	Well	Depth (m)	TOC (%)	Porosity (%)	Clay mineral (%)	Analcime (%)	Quartz (%)	Feldspar (%)	Calcite (%)	Dolomite (%)	Pyrite (%)
Weiyuan area	A1	2647.33	4.5	6.1	26.6	1.7	37.1	4.6	11.8	13.5	4.7
		2654.23	2.6	3.4	21.7	0.9	43.5	8.2	9.0	12.0	4.5
		2658.58	2.5	4.9	17.9	1.2	51.2	8.1	11.2	7.8	2.5
		2663.64	3.5	4.5	5.4	0.8	67.0	1.5	17.5	4.0	3.8
	A2	2837.98	4.8	6.2	30.4	0.1	31.5	4.8	19.9	11.1	2.2
		2846.48	2.9	4.5	20.4	0.0	51.9	6.7	7.4	9.6	4.0
		2852.86	3.0	4.9	23.2	0.0	49.4	10.0	9.3	5.3	2.8
		2856.79	4.3	8.1	6.5	0.1	65.9	1.1	18.8	6.4	1.2
	B1	2726.56	3.6	7.5	31.0	0.0	30.9	4.0	21.8	10.7	1.6
		2738.51	2.3	6.5	26.0	0.0	44.9	8.1	10.7	5.7	4.6
		2742.54	3.0	6.2	26.2	0.0	46.3	8.5	11.6	4.7	2.7
		2747.56	4.8	5.6	12.1	0.0	70.3	1.8	10.4	4.6	0.8
	B2	2932.52	5.0	9.9	39.7	0.0	34.1	6.3	10.0	6.2	3.7
		2940.33	2.9	7.6	31.0	0.8	47.5	10.8	4.1	3.1	2.7
		2943.20	2.8	7.2	26.7	0.0	48.8	9.5	9.0	3.7	2.3
		2946.27	5.5	7.3	18.4	0.0	39.8	3.3	26.7	8.8	3.0
Luzhou area	X1	4095.28	2.8	5.7	37.0	0.0	38.0	11.0	5.0	5.0	4.0
		4104.68	4.5	6.7	29.0	0.0	50.0	7.0	3.0	6.0	5.0
		4108.77	5.3	6.8	17.0	0.0	67.0	3.0	4.0	5.0	4.0
		4113.69	5.1	6.8	16.0	0.0	60.0	3.0	9.0	8.0	4.0
	Y1	4132.95	2.7	3.8	40.0	0.0	35.0	9.0	7.0	6.0	3.0
		4139.78	3.7	6.2	32.0	0.0	46.0	6.0	3.0	6.0	7.0
		4144.76	5.0	5.0	21.0	0.0	56.0	4.0	8.0	6.0	5.0
		4149.73	4.4	4.2	23.0	0.0	33.0	4.0	28.0	10.0	2.0
	Y2	4107.02	2.3	3.8	34.0	0.0	35.0	10.0	7.0	10.0	4.0
		4116.93	4.4	4.5	21.0	0.0	47.0	2.0	6.0	13.0	11.0
		4120.10	3.7	5.2	12.0	0.0	49.0	3.0	20.0	12.0	4.0
		4123.53	7.8	4.0	8.0	0.0	78.0	3.0	4.0	5.0	2.0
	Y4	3767.04	2.3	4.8	46.0	0.0	28.0	12.0	7.0	4.0	3.0
		3776.00	3.1	5.5	35.0	0.0	37.0	8.0	3.0	13.0	4.0
		3781.75	4.8	5.9	20.0	0.0	57.0	4.0	5.0	10.0	4.0
		3785.20	4.3	5.3	22.0	0.0	60.0	4.0	4.0	6.0	4.0

### Shale pore morphology characteristics

**Figure 3:**
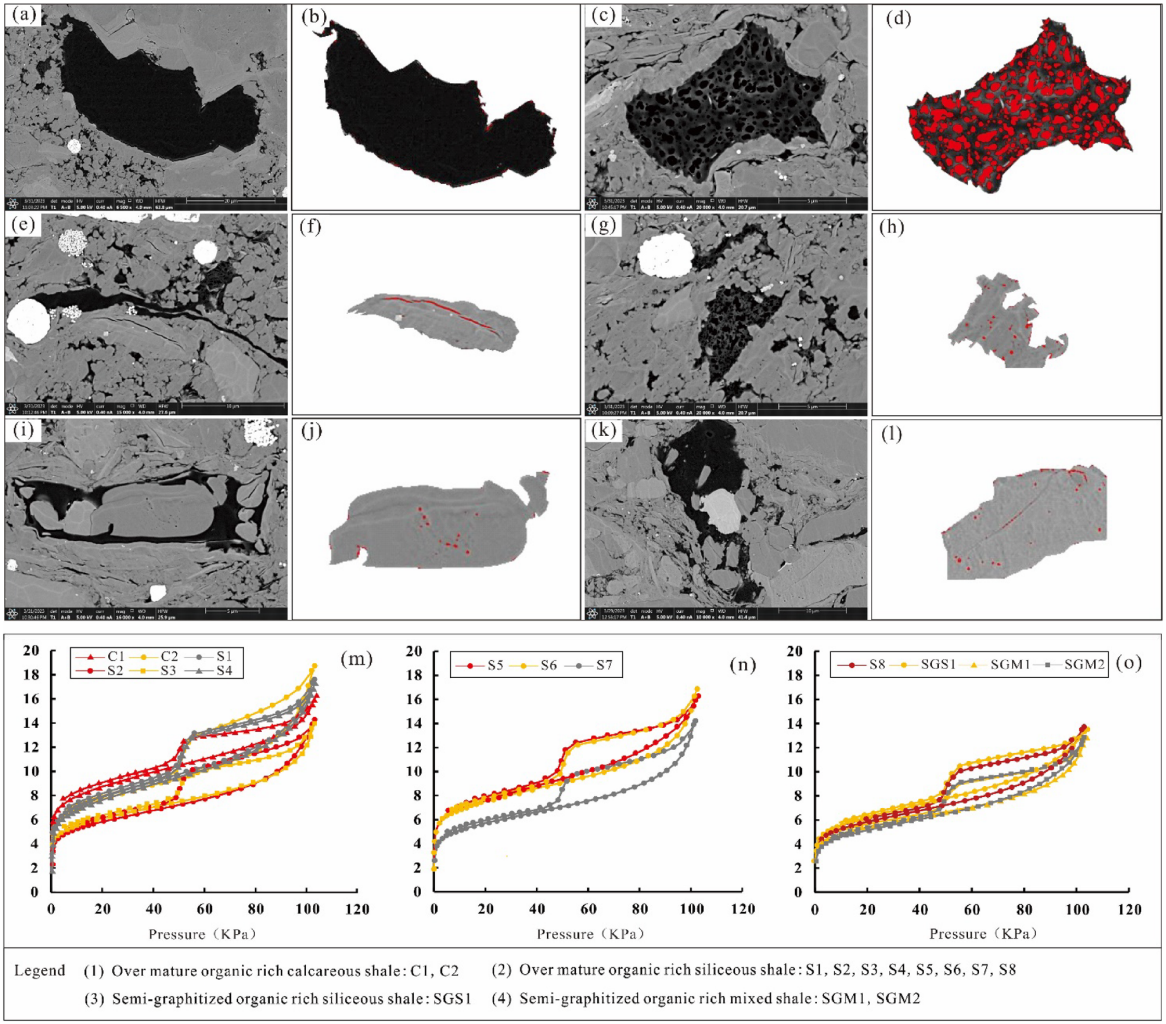
Comparison of the pore types and nitrogen adsorption–desorption isotherm curves of different types of gas wells in the Longyi_1_ sub-member in the Luzhou area. (**a,b**) Well Y1, 4150.21 m, solid kerogen pores, (**c,d**) Well X1, 4105.95 m, pyrobitumen pores, (**e,f**) Well X1, 4112 m, inner pores of clay mineral, (**g,h**) Well X1, 4112 m, intergranular pores of siliceous minerals, (**i,j**) Well X1, 4112 m, dissolution pores of siliceous minerals, (**k,l**) Well X1, 4112 m, carbonate dissolution pores.

Image software is used to process the images, grayscale processing is performed on the SEM images of the shale in the study area, and the basic values of pores are extracted and calculated to obtain representative pore structure parameters(Fig. 3a–l). SEM with argon ion beam milling shows that the inorganic pores of the reservoir in the study area are mainly intragranular dissolution pores, and the intergranular pores are less developed^19^. The organic matter pores are in the growth period, and the pores are well preserved; most of them are isolated round pores, and the size of organic matter pores is mainly 100–200 nm.

The hysteresis loop characteristics of N_2_ adsorption in the samples in the study area are obvious. The shapes of the hysteresis loops of the three gas wells correspond to the H_2_ type, indicating that all are ink bottle-type pores (Fig. 3a–l). The hysteresis loop area of the overmature organic-rich calcareous shale of the well Y1 in sublayer 1 of Longyi_1_ is small, indicating that the space of ink bottle-type pores in this reservoir facies is small, the space of ink bottle-type pores of the reservoir facies in other sublayers is similar, and the adsorption capacity of overmature organic-rich siliceous shales in sublayers 2–3 is better than that in sublayer 1 (Fig. 3m). The hysteresis loop area of the overmature organic-rich siliceous shale of the well Y2 in sublayer 1 of Longyi_1_ is smaller than that in sublayers 2 and 3, indicating that the ink bottle-type pore space is smaller than that of sublayers 2 and 3; however, the adsorption capacities of the overmature organic-rich siliceous shale in sublayers 1 and 2 are better than those of sublayer 3 (Fig. 3n). The space of semi-graphitized organic-rich mixed shale of the well Y3 in sublayer 3 in the 1st sub-member is smaller than that of sublayers 1 and 2; the adsorption capacity of semi-graphitized organic-rich mixed shale in sublayer 3 is lower than that of sublayers 1 and 2 (Fig. 3o).

### Quantitative characterization of various types of shale pores

Shale pores are divided into two types: organic pores and inorganic pores. Inorganic pores are further divided into intergranular pores, intercrystalline pores, intragranular dissolution pores, and interlayer fractures of clay minerals^26^. Organic matter pores are the most important in shale gas reservoirs, and they comprise the main storage space and seepage channels in shale; the complex internal structure greatly increases the shale pore volume and pore surface area. According to the SEM images, the shale pores developed in the Longyi_1_ sub-member in the study area are divided into six categories: kerogen pores (dominant), pyrobitumen pores, clay mineral intragranular pores, siliceous mineral intergranular pores, siliceous mineral dissolution pores, and carbonate dissolution pores^27^. The porosities of the pyrobitumen pores are the highest, reaching 21.04%–31.65%, while the porosities of solid kerogen pores, siliceous mineral dissolution pores, and carbonate dissolution pores are low, ranging from 0.48 to 1.80%. A quantitative analysis of the contribution ratios of each matrix component to the shale pore space shows that the organic matter pores mainly contribute to the pore development of shale reservoirs, ranging from 59.99 to 63.39%, of which 99% are pyrobitumen pores.

**Figure 4:**
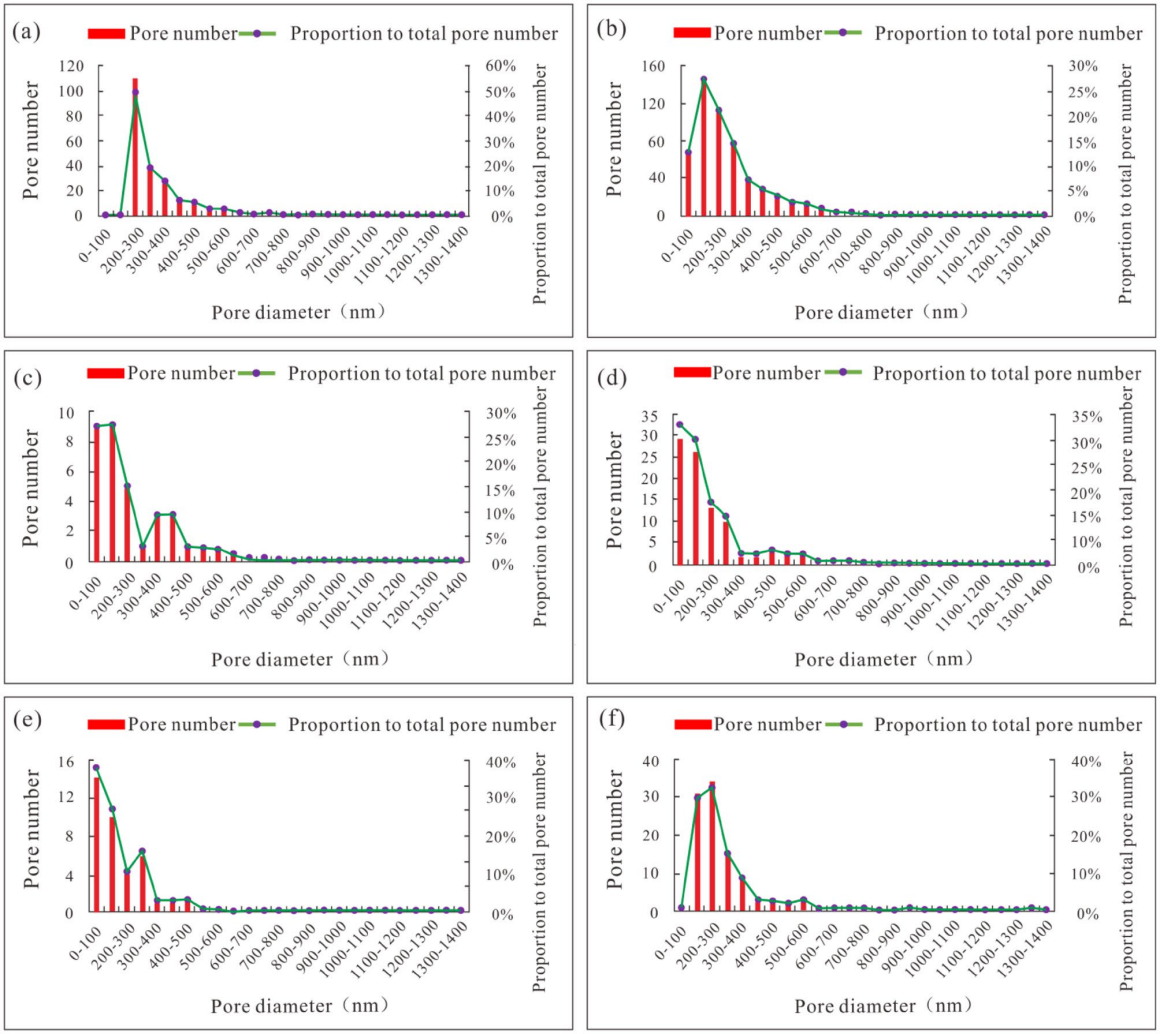
Pore size distribution characteristics of pores with different matrices of the Longyi_1_ submember in the Well Y1. (**a**) Pore aperture of solid kerogen pores, (**b**) pore aperture of pyrobitumen pores, (**c**) pore aperture of inner pores of clay mineral, (**d**) pore aperture of intergranular pores of siliceous minerals, (**e**) pore aperture of dissolution pores of siliceous minerals, (**f**) pore aperture of carbonate dissolution pores.

The various types of pores in different gas wells in the Longyi_1_ sub-member are analyzed to obtain the pore size and average surface porosity values of shale pores with different matrices. The main distribution range of the solid kerogen pore size is 200–500 nm (Fig. 4a). The main distribution range of the pyrobitumen pore size is 2–500 nm (Fig. 4b). The main distribution range of the clay mineral intergranular pore size is 2–300 nm (Fig. 4c). The main distribution range of the siliceous mineral intergranular pore size is 2–400 nm (Fig. 4d). The main distribution range of the siliceous mineral dissolution pore size is 2–300 nm (Fig. 4e). The main distribution range of the carbonate dissolution pore size is 100–400 nm (Fig. 4f).

**Figure 5:**
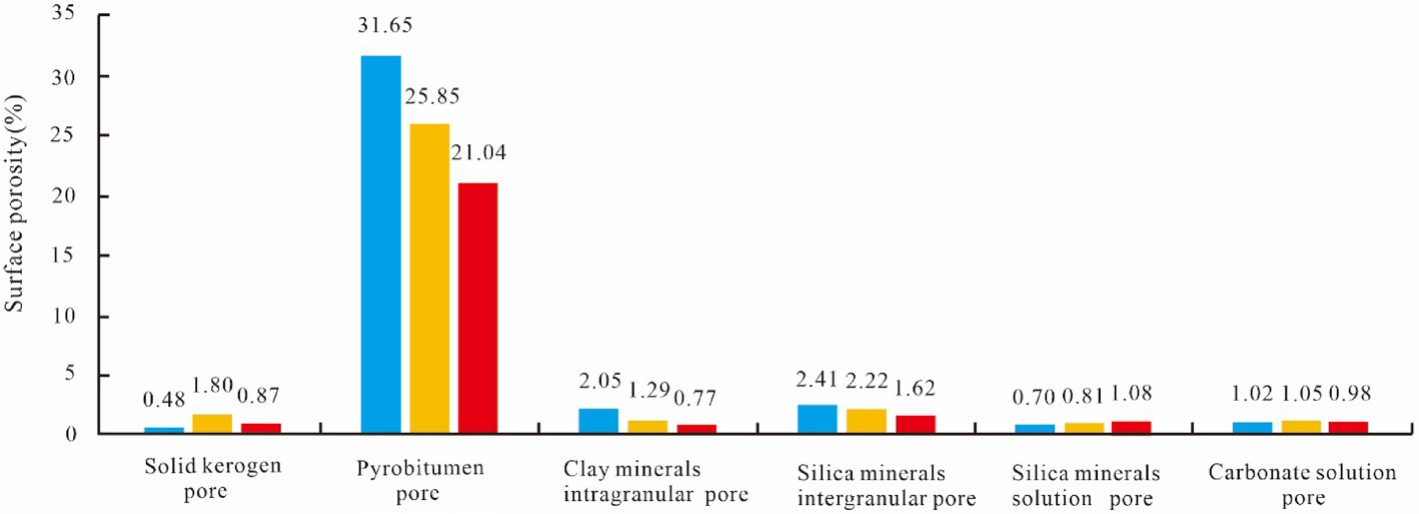
Comparison of the surface porosities between different types of shale gas wells in the Longyi_1_ sub-member in the Luzhou area.

Table 3 shows the SEM image processing results of different types of shale pores in various gas wells in the Longyi_1_ sub-member. In each type of gas well, (1) for pyrobitumen pores, the number of pores is large, the roundness is good, the pore size distribution range is large, the area of a single pore is relatively large, and the surface porosity is high, causing pyrobitumen pores to contribute most of the storage space. (2) For siliceous mineral intergranular pores, the number of pores is large, and the pore size is small, and for clay mineral intergranular pores, the number of pores is small, and the complexity is high, resulting in medium surface porosity; these two pore types contribute to some of the reservoir space. (3) For solid kerogen pores, the number of pores is small, the pore size is small, and the area of a single pore is small. For siliceous mineral dissolution pores, the number of pores is small, and the pore size is small. For carbonate dissolution pores, the number of pores is mid-level, and the pore size is small. The surface porosities of these three pore types are the lowest (Fig. 5). For the pyrobitumen pores, clay mineral intergranular pores, and siliceous mineral intergranular pores, the surface porosity follows the order of the Well Y1 > the Well Y2 > the Well Y3 (Table 3).

**Table 3:** SEM image processing results of different types of shale pores in different gas wells in the Longyi_1_ sub-member in the Luzhou area^45,46^.

Well	Pore type	Plane porosity (%)	The main distribution range of aperture (nm)
Y1	Solid kerogen pore	0.48	200–500
	Pyrobitumen pore	31.65	2–500
	Clay mineral intergranular pore	2.04	2–300
	Siliceous mineral intergranular pore	2.41	2–400
	Siliceous mineral dissolution pore	0.70	2–300
	Carbonate dissolution pore	1.02	100–400
Y2	Solid kerogen pore	1.80	200–500
	Pyrobitumen pore	25.85	2–400
	Clay mineral intergranular pore	1.29	2–600
	Siliceous mineral intergranular pore	2.22	2–400
	Siliceous mineral dissolution pore	0.81	2–400
	Carbonate dissolution pore	1.05	100–500
Y3	Solid kerogen pore	0.87	2–200
	Pyrobitumen pore	21.04	2–500
	Clay mineral intergranular pore	0.77	200–400
	Siliceous mineral intergranular pore	1.62	2–300
	Siliceous mineral dissolution pore	1.08	2–300
	Carbonate dissolution pore	0.98	100–400

### Quantitative characterization of the shale pore structure

In this study, the data from the high-pressure mercury intrusion experiment are used to characterize the macropores, the data from the nitrogen adsorption experiment are used to characterize the mesopores and the data from the CO_2_ adsorption experiment are used to characterize the micropores^28,29^. Therefore, using these three methods, we can achieve the characterization of all pore diameters of shale.

**Figure 6:**
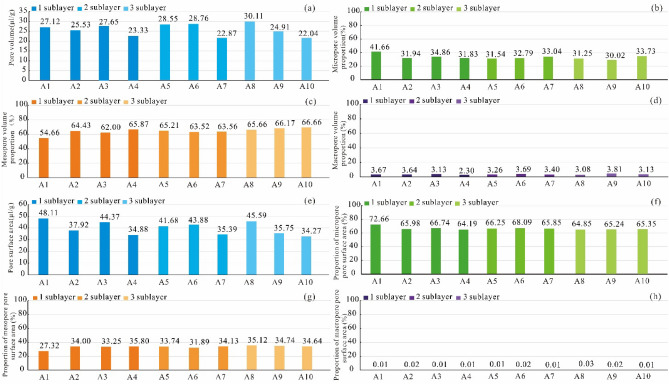
Pore volume characteristics and pore specific surface area characteristics of the shale reservoirs of each reservoir facies in the Longyi_1_ submember in the Luzhou area. (**a**) Pore volume of shale reservoirs, (**b**) the proportion of micropore volume to total pore volume, (**c**) the proportion of mesopore volume to total pore volume, (**d**) the proportion of macropore volume to total pore volume, (**e**) specific surface area, (**f**) the proportion of micropore specific surface area to total pore specific surface area, (**g**) the proportion of mesopore specific surface area to total pore specific surface area, (**h**) the proportion of macropore specific surface area to total pore specific surface area.

By taking samples in the Longyi_1_ submember as an example, the pore volume and pore surface area distribution histograms are obtained by three types of experiments, and the proportion of micropores, mesopores, and macropores in terms of pore volume and surface area are calculated. The shale pore volume of each reservoir facies in the Longyi_1_ submember is 22–30 μL/g (Fig. 6a). The pore volume of the shale reservoir in sublayer 1 ranges from 23.33 to 27.65 μL/g, with an average value of 25.91 μL/g. The pore volume of the shale reservoir in sublayer 2 ranges from 22.87 to 28.76 μL/g, with an average value of 26.72 μL/g. The pore volume of the shale reservoir in sublayer 3 is the lowest, ranging from 22.04 to 30.11 μL/g, with an average value of 25.69 μL/g. The pore volume of each reservoir facies is mainly provided by mesopores (54–66%), followed by micropores (31–41%) (Fig. 6a–d).

The pore specific surface area of the shale reservoir in sublayer 1 ranges from 34.88 to 48.11 m^2^/g, with an average value of 41.32 m^2^/g; The pore specific surface area of the shale reservoir in sublayer 2 ranges from 35.39 to 43.88 m^2^/g, with average value is 40.32 m^2^/g; the pore specific surface area of shale reservoir in sublayer 3 is the lowest, ranging from 34.27 to 45.59 m^2^/g, with average value is 38.53 m^2^/g. For each reservoir facies, micropores contribute the most to the pore specific surface area (64–72%), followed by mesopores (27–35%) (Fig. 6e–h).

## Results and discussion

### Analysis of factors affecting the development of shale pore structure

#### Relationships between the pore structure parameters, TOC contents, and the degree of thermal evolution

**Figure 7:**
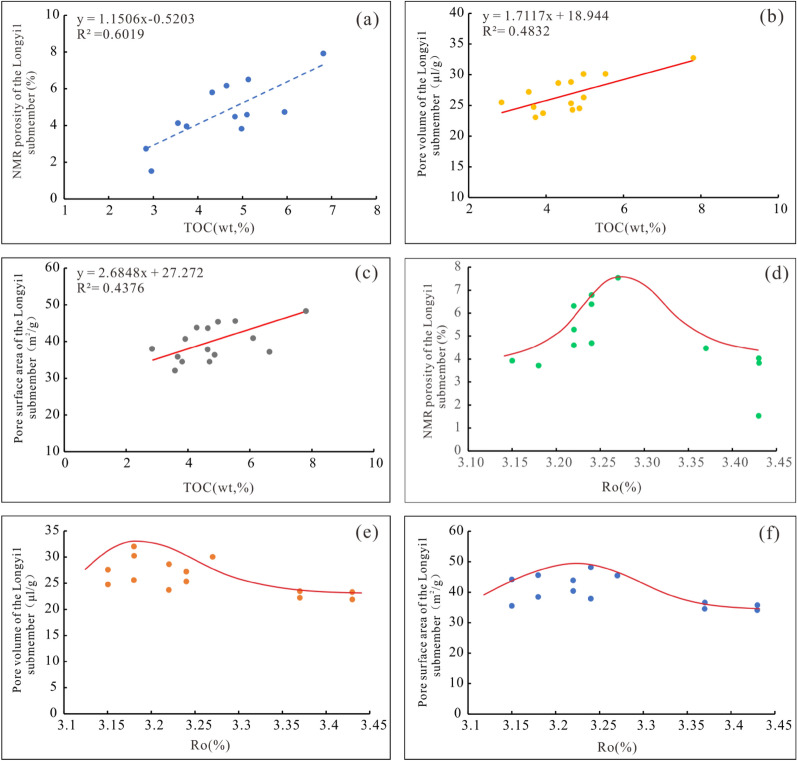
Correlation analysis of the TOC content, Ro, and pore parameter values in the Longyi_1_ sub-member in the Luzhou area. (**a**) Correlation analysis between TOC content and porosity, (**b**) correlation analysis between TOC content and pore volume, (**c**) correlation analysis between TOC content and pore specific surface area, (**d**) correlation analysis between Ro and porosity, (**e**) correlation analysis between Ro and pore volume, (**f**) correlation analysis between Ro and pore specific surface area.

The TOC content of marine shale in the southern Sichuan is high. For kerogen, type II is dominant, with a small amount of type II1, and the maturity is relatively high. The TOC content is positively correlated with the porosity, pore volume, and pore specific surface area (Fig. 7a–c). Organic pores mainly contribute to the development of pores in shale reservoirs. Organic matter includes kerogen and pyrobitumen. With the increase in thermal evolution, kerogen first generates oil, and crude oil enters the inorganic pores composed of rigid minerals, such as quartz, to form pyrobitumen. Due to the oil and gas formed by thermal evolution, both kerogen and pyrobitumen can form pores; however, the kerogen pores are strongly compacted under the action of the overlying pressure, the pores are small, the roundness is low, and the connectivity is poor, while the pyrobitumen pores are effectively preserved by the support of rigid minerals, with relatively large pore sizes, high roundness, and good connectivity. Pyrobitumen pores are the main type of developed organic pores.

**Figure 8:**
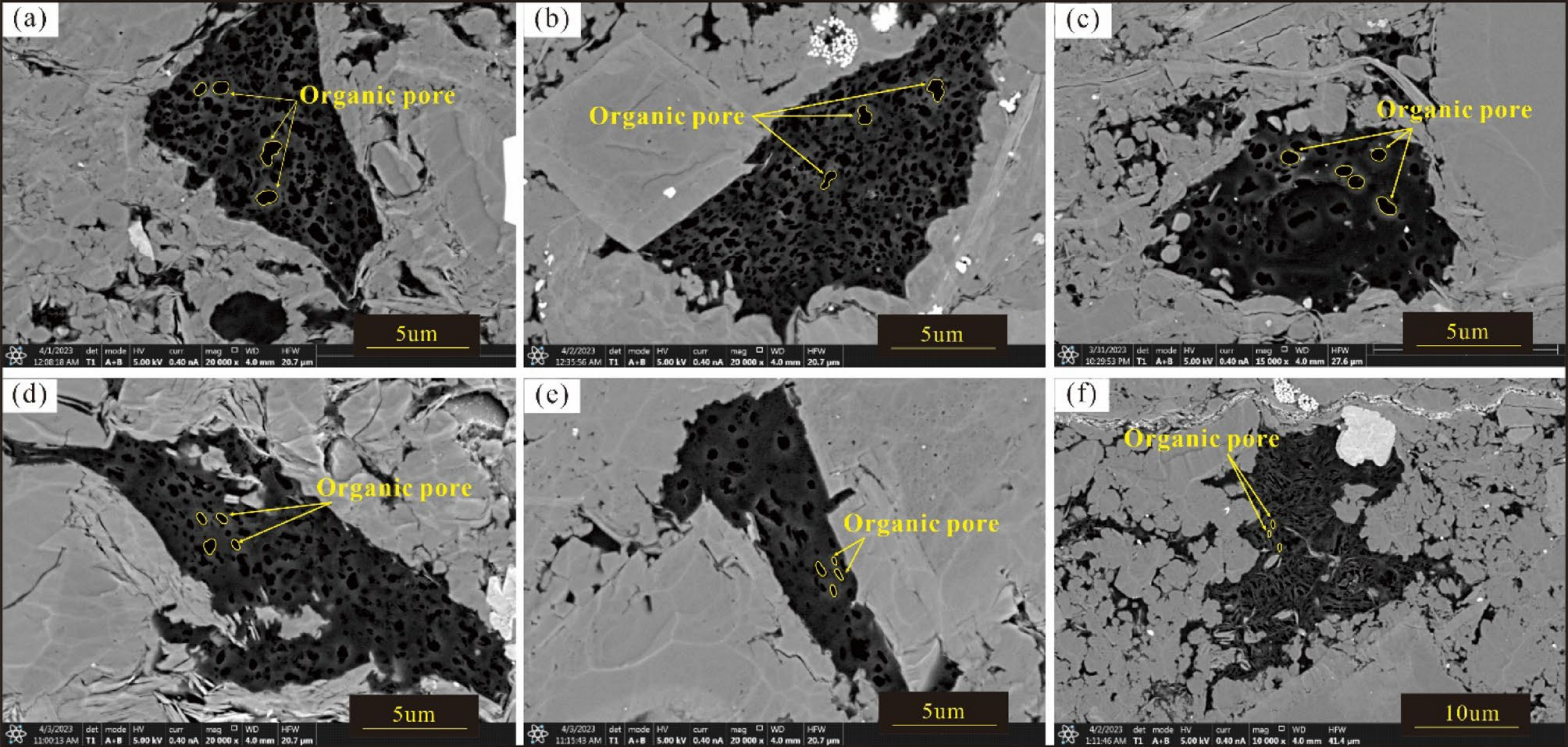
Development characteristics of organic matter pores in samples of the Longyi_1_ sub-member in the Luzhou area. (**a**) Well Y1, 4142.15 m, Ro = 3.26%, (**b**) Well Y4, 3784.17 m, Ro = 3.10%, (**c**) Well X1, 4105.95 m, Ro = 3.17%, (**d**) Well Y8, 4096.94 m, Ro = 3.37%, (**e**) Well Y8, 4094.72 m, Ro = 3.37%.

The Longyi_1_ sub-member in the study area is in the overmature stage. With increasing Ro, the porosity increases (Fig. 7d), and the pore volume and specific surface area reach their maximum values (Fig. 7e, f). In the semi-graphitization stage, with increasing Ro, the porosity decreases, and the pore volume and pore specific surface area gradually decrease. The SEM with argon ion beam milling shows that organic matter pores in the overmature stage have good roundness, large pore sizes, and good connectivity (Fig. 8a–c); in the semi-graphitization stage, the organic matter pores have poor roundness, small pore sizes, and mainly exist as single pores (Fig. 8d–f). When the shale Ro is between 2.0 and 3.3%, the organic matter generates a large amount of dry gas, and the organic matter pores are developed in large quantities; many pyrobitumen pores with good roundness and good connectivity are dominant. When the shale Ro is higher than 3.3% (Fig. 8d–f), the organic matter begins to graphitize gradually, the number of organic matter pores decreases, the roundness decreases, and the surface porosity decreases, thereby decreasing the shale storage capacity and pore connectivity.

#### Relationship between the shale mineral compositions and pore structure parameters

**Figure 9:**
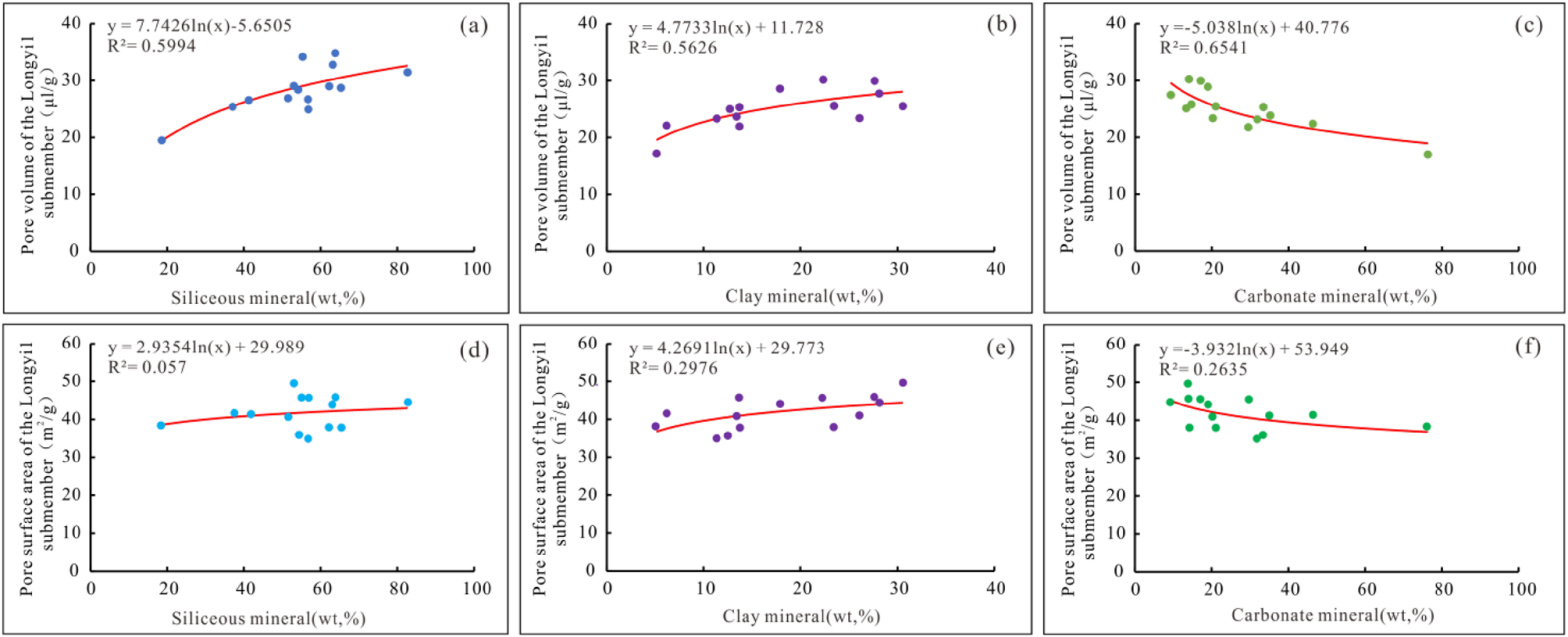
Correlation analysis of the inorganic components and pore parameters in the Longyi_1_ sub-member in the Luzhou area. (**a**) Correlation analysis between siliceous mineral content and pore volume, (**b**) correlation analysis between clay mineral content and pore volume, (**c**) correlation analysis between carbonate mineral content and pore volume, (**d**) correlation analysis between siliceous mineral content and pore specific surface area, (**e**) correlation analysis between clay mineral content and pore specific surface area, (**f**) correlation analysis between carbonate mineral content and pore-specific surface area.

The mineral compositions of shale reservoirs control the pore volume and pore specific surface area values to a certain extent^19^. Correlations of siliceous, clay, and carbonate mineral contents with the pore volume and pore specific surface area values in the Longyi_1_ sub-member are plotted. Siliceous minerals are positively correlated with the pore volume and weakly negatively correlated with the pore specific surface area (Fig. 9a, d), indicating that with the increase in the siliceous mineral content, the pore volume of shale in the Longyi_1_ sub-member increases, while the pore specific surface area has no obvious trend. Clay minerals are weakly correlated with pore volume and positively correlated with the pore specific surface area (Fig. 9b, e); carbonate minerals are not correlated with the pore volume and pore specific surface area (Fig. 9c, f).

Pore types of siliceous mineral intragranular pores, clay mineral intragranular pores, carbonate intragranular pores, other intergranular pores, and dissolution pores are developed. The siliceous mineral content is high, but due to the influence of diagenesis, the numbers of intergranular pores and dissolution pores developed are small, and the surface porosity is low; thus, siliceous mineral pores can provide a small amount of pore volume for free gas. Siliceous minerals have a small specific surface area and weak adsorption capacity values, so they cannot provide storage space for adsorbed gas. The content of clay minerals is low, and due to the influence of diagenesis, the number of intragranular pores developed is small, and the surface porosity is low; thus, clay mineral pores can provide a small amount of pore volume for free gas. Clay minerals have certain specific surface areas per unit and have certain adsorption capacities for methane; thus, clay mineral pores can provide some storage space for adsorbed gas. The content of carbonate minerals is small, the number of dissolution pores is small, the surface porosity is low, the specific surface area is small, and the adsorption capacity is weak; thus, so carbonate minerals have no significant impact on the shale storage capacity.

### Controlling effects of shale pores on gas-bearing properties

**Figure 10:**
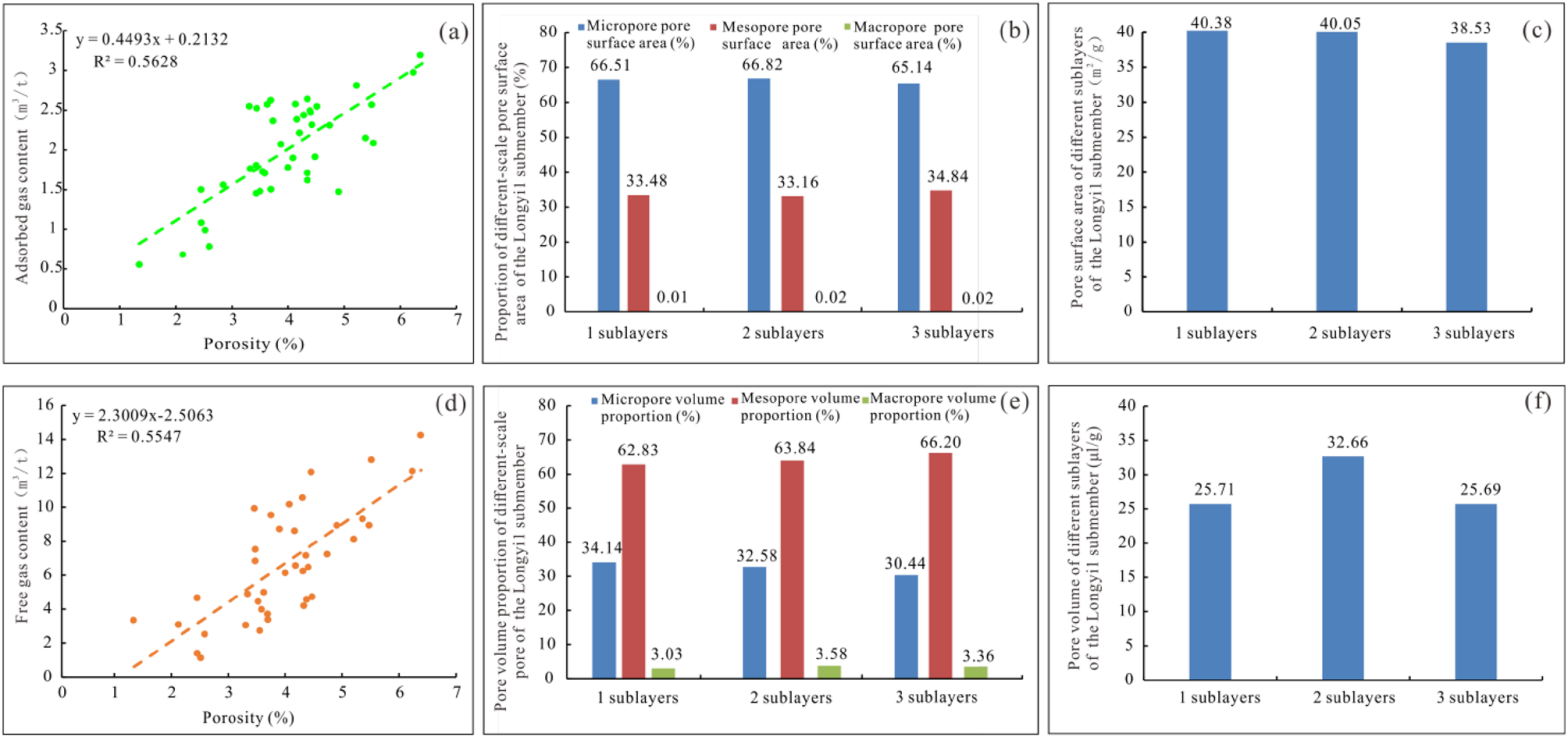
Correlation analysis of the adsorbed gas and pore parameters in the Luzhou area. (**a**) Relationship between the adsorbed gas and porosity of the Longyi_1_ sub-member, (**b**) proportions of the specific surface areas of pores with different sizes in each sublayer, (**c**) specific surface areas of pores with different sizes in each sublayer, (**d**) relationship between free gas and porosity in the Longyi_1_ sub-member, (**e**) proportions of the pore volumes of pores with different sizes in each sublayer. (**f**) proportions of the pore volumes of pores with different sizes in each sublayer.

Adsorbed gas and free gas are the main components of shale gas. Adsorbed gas is mainly adsorbed on organic matter and minerals surfaces, and free gas mainly occurs in the shale pores^30^. Therefore, the content of adsorbed gas is mainly related to the specific surface area of shale, and the amount of free gas is mainly related to the pore volume of shale. The shale adsorbed gas in the Longyi_1_ sub-member mainly occurs on the surfaces of micropores, followed by mesopores, and macropores have very small pore specific surface areas (Fig. 10a)^31,32^. In sublayer 1, the average pore specific surface areas of micropores, mesopores, and macropores account for 66.82%, 33.16%, and 0.02%, respectively. In sublayer 2, the average pore specific surface areas of micropores, mesopores, and macropores account for 66.51%, 33.48%, and 0.01%, respectively. In sublayer 3, the average pore specific surface areas of micropores, mesopores, and macropores account for 65.14%, 34.84%, and 0.02%, respectively (Fig. 10b). The average pore specific surface areas of sublayers 1, 2, and 3 are 40.38 m^2^/g, 40.05 m^2^/g, and 38.53 m^2^/g (Fig. 10c), respectively. Sublayers 1, 2, and 3 have similar specific surface areas, with sublayer 1 slightly higher than that of sublayers 2 and 3.

The shale free gas in the Longyi_1_ sub-member mainly occurs in mesopores, followed by micropores, and macropores have very small pore volumes(Fig. 10d). In sublayer 1, the average pore volumes of the mesopores, micropores, and macropores account for 62.83%, 34.14%, and 3.03%, respectively. In sublayer 2, the average pore volumes of the mesopores, micropores, and macropores account for 63.84%, 32.58%, and 3.58%, respectively. In sublayer 3, the average pore volumes of the mesopores, micropores, and macropores account for 66.20%, 30.44%, and 3.36%, respectively (Fig. 10e). The average pore volumes of sublayers 1, 2, and 3 are 25.71 μL/g, 32.66 μL/g, and 25.69 μL/g, respectively (Fig. 10f). The average pore volume of sublayer 2 is the largest, and the average pore volumes of sublayers 1 and 3 are similar and smaller than that of sublayer 2.

### Evolution processes of the conditions for controlling shale gas reservoir

**Figure 11:**
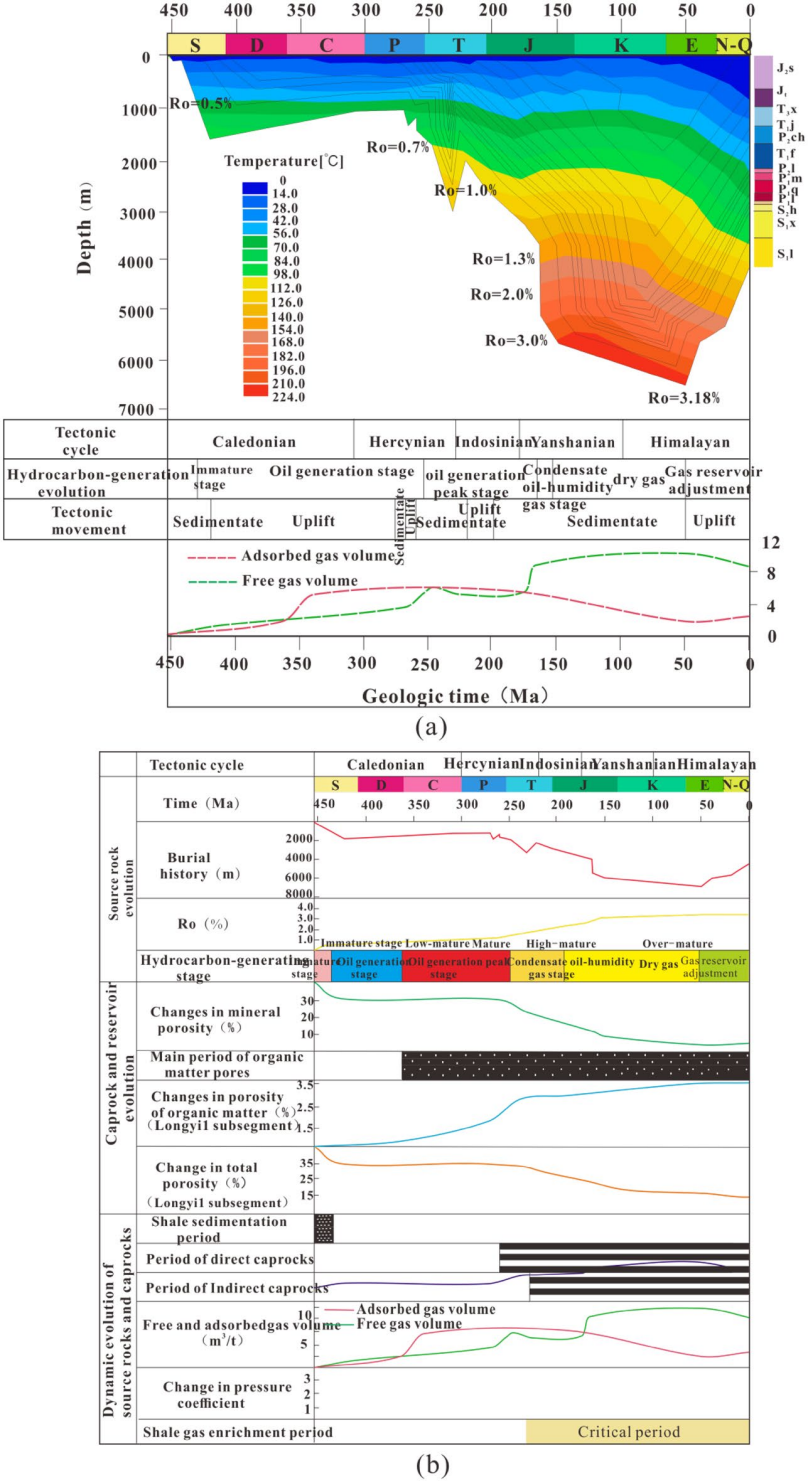
Matching relationship of the evolution process of shale gas source-reservoir-cap reservoir-controlling conditions in Well Y1 of the Longyi_1_ sub-member in the Luzhou area. (**a**) The relationship between the structural evolution of Longyi_1_ sub-member and the evolution of shale gas bearing properties in Well Y1, (**b**) matching relationship of the evolution process of "source reservoir cap" control conditions for shale gas in the Longyi_1_ sub-member in Well Y1 (this figure is generated in CorelDRAW 2020 software, https://www.coreldrawchina.com/).

By comprehensively using the burial history of the Luzhou area, combined with the adsorption gas prediction model and the free gas prediction model, the evolution pattern of the occurrence state of shale gas in the Luzhou area under tectonic control is restored (Fig. 11a). Using the erosion thickness and the recently measured Ro as constraints, the burial history and thermal evolution history of the Longyi_1_ sub-member in the study area are restored. By taking the Well Y1 as an example, the relationships between the tectonic evolution characteristics and gas-bearing properties are analyzed. The shale in the Longyi_1_ sub-member has undergone four evolution stages. (1) Early- to mid-Silurian immature stage: due to the influence of the early Caledonian orogeny, the strata subside stably at this stage, and the shale Ro is less than 0.5%. Only a very small amount of oil and gas is generated in the immature stage. (2) Late Silurian–Middle Jurassic oil-generating stage: the strata enter the low-mature stage in the late Silurian, and oil generation starts. In the middle Permian–Jurassic, affected by the Variscan orogeny, Indo-China Movement, and Yanshan Movement, stratum subsidence and uplift are interlaced, and the overall process is dominated by rapid deposition. Shale enters the peak of oil generation in the Triassic period, and oil and gas migrate to the surrounding rock. In the middle Jurassic, shale enters the high maturity stage; from there, gas condensate and wet gas start to generate, the burial depth of the formation increases, and the content of adsorbed gas increases. In this stage, the adsorbed gas is dominant, generating only a small amount of free gas. (3) The Late Jurassic–early Paleogene gas generation stage is successively affected by the Yanshan and the early Himalayan movements. The strata continue to subside, the burial depth continues to increase, the inhibitory effect of formation pressure on adsorbed gas exceeds the promotional effect of formation temperature, the amount of adsorbed gas decreases while the free gas content increases, many liquid hydrocarbons are pyrolyzed to produce dry gas, the shale maturity increases to a maximum value of 3.18%, the amount of adsorbed gas continues to decrease, and the amount of free gas continues to increase. (4) Late Paleogene–Neogene–Quaternary gas reservoir adjustment stage: affected by the late Himalayan movement, the stratum is gradually uplifted. With the decrease in the stratum burial depth, free gas content decreases slowly, and the content of adsorbed gas increases slowly.

Porosity evolution: for Well Y1 in the Longyi_1_ sub-member, the porosities of shale mineral pores decrease with increasing burial depth in the early Silurian after shale deposition and remain almost unchanged during the Late Silurian–Late Permian. From the Early Triassic to the middle of the Paleogene, the burial depth increases to its maximum value, and the porosities of shale mineral pores decrease to the minimum value. The porosities of organic matter pores increase rapidly from the high-mature wet gas-generating stage in the early Carboniferous and remain almost unchanged after reaching the maximum burial depth and maturity. The variation trend of total porosity is similar to that of mineral pores, and the total porosity is slightly larger than that of mineral pores. Evolution of caprock: the direct caprock is formed from the wet gas-generating stage in the late Permian to the present. During this period, with increasing burial depth, the porosity and permeability decrease, and a good direct caprock is formed when many organic pores are formed in the shale. The gypsum-salt rocks deposited in the Middle-Late Triassic provide a good indirect caprock for the shale of the Longmaxi Formation. Variation in pressure coefficient: With the pyrolysis of organic matter to generate oil and gas through the sealing of direct caprock and indirect caprock, the formation pressure coefficient increases gradually with the increase in burial depth; the coefficient reaches the highest value in the early Paleogene and then gradually decreases with the stratum uplift (Fig. 11b).

### Effects of 3D effective sealing on the gas-bearing properties of shale

#### Horizontal shale bedding closure

According to the relative shale bedding sealing pressure size and the shale bedding surface pressure, the sealing property is determined. According to the difference in mineral composition, the compressive strength of shale is generally between 19.61 and 68.65 MPa, but the strength of siliceous shale is slightly increased. When the vertical pressure on the shale bedding surface is greater than the compressive strength, the shale bedding surface is closed^33^. In the Weiyuan area, the Wufeng–Longmaxi Formation shale in Well A3 is buried at a depth of approximately 2573 m. The vertical stress of the formation is 58.0 MPa, which is slightly lower than the shale bedding sealing pressure, the pressure coefficient is 1.40, and the single-well productivity is 2.75 × 10^4^ m^3^/day. The Wufeng–Longmaxi Formation shale in Well B1 is buried at a depth of approximately 2749 m, the vertical pressure of the formation is 69.8 MPa, the formation pressure coefficient is 1.58, and the single-well production is 22.73 × 10^4^ m^3^/day. The Wufeng–Longmaxi shale in Well B3 is buried at a depth of approximately 3355 m, the vertical pressure of the formation is 82.32 MPa, the formation pressure coefficient is 1.96, and the single-well production is 21.29 × 10^4^ m^3^/day. In the Luzhou area, the Wufeng–Longmaxi Formations shale in Well X2 is buried at a depth of approximately 3815 m, the vertical stress is 101.3 MPa, the formation pressure coefficient is 1.82, which is greater than the sealing pressure of the shale bedding, and the test productivity is 137.9 × 10^4^ m^3^/day. The vertical pressure of Well X3 is 105.05 MPa, the formation pressure coefficient is 2.36, and the tested productivity is 30.55 × 10^4^ m^3^/day. The vertical pressure of Well Y1 is 103.68 MPa, the formation pressure coefficient is 2.17, and the tested productivity is 32.08 × 10^4^ m^3^/day. A comparison of the single-well productivity of wells with different burial depths in the Weiyuan area and Luzhou area shows that when the vertical pressure of the formation is close to or greater than the compressive strength of the rock, the single-well productivity of shale gas is high, indicating that the high horizontal shale bedding closure is conducive to the preservation of shale gas.

#### Roof and floor closure

The formation pressure coefficient of Well B1 is 1.58, the average gas content in organic-rich shale is 3.24 m^3^/t, and the porosity is 6.70%. The good roof and floor sealing properties arise due to the large differences in physical properties between the roof and floor, and the organic-rich shale is an important reason for the high gas-bearing capacity of organic-rich shale^19,34,35^. The average porosities of the roof, regional caprock, and floor are approximately 5.35%, 2.50%, and 1.20%, respectively, and all are quite different from that of the organic-rich shale at approximately 6.70%. The formation pressure coefficient of Well X3 is 2.36, the average gas content of organic-rich shale is 3.46 m^3^/t, and the porosity is 5.44%. The average porosities of the roof, regional caprock, and floor are approximately 2.31%, 1.10%, and 1.45%, respectively, and all are quite different from the organic-rich shale at approximately 5.44% (Table 4).

**Table 4:** Lithology, porosity, and thickness characteristics of the shale, roof, floor, and regional caprock in Wells B1 and X3.

Name	Formation	Lithology	Average porosity of Well B1 (%)	The thickness of Well B1 (m)	Average porosity of Well X3 (%)	The thickness of Well X3 (m)
Regional caprock	Lower Triassic Jialingjiang Formation	Gypsum rock salt	1.00	> 50	1.00	> 50
Roof	2nd member of the Lower Silurian Longmaxi Formation	Lime shale	1.46	45	1.46	45
	The upper part of the 1st member of the Lower Silurian Longmaxi Formation	Silty shale	1.69	97	1.69	97
Shale	Upper Ordovician Wufeng Formation–Lower Silurian Longmaxi Formation	Organic-rich siliceous shale	4.00	46	4.00	46
Floor	Upper Ordovician Linxiang Formation	Nodular limestone	1.45	> 35	1.45	> 35
	Middle Ordovician Pagoda Formation	Limestone				

**Figure 12:**
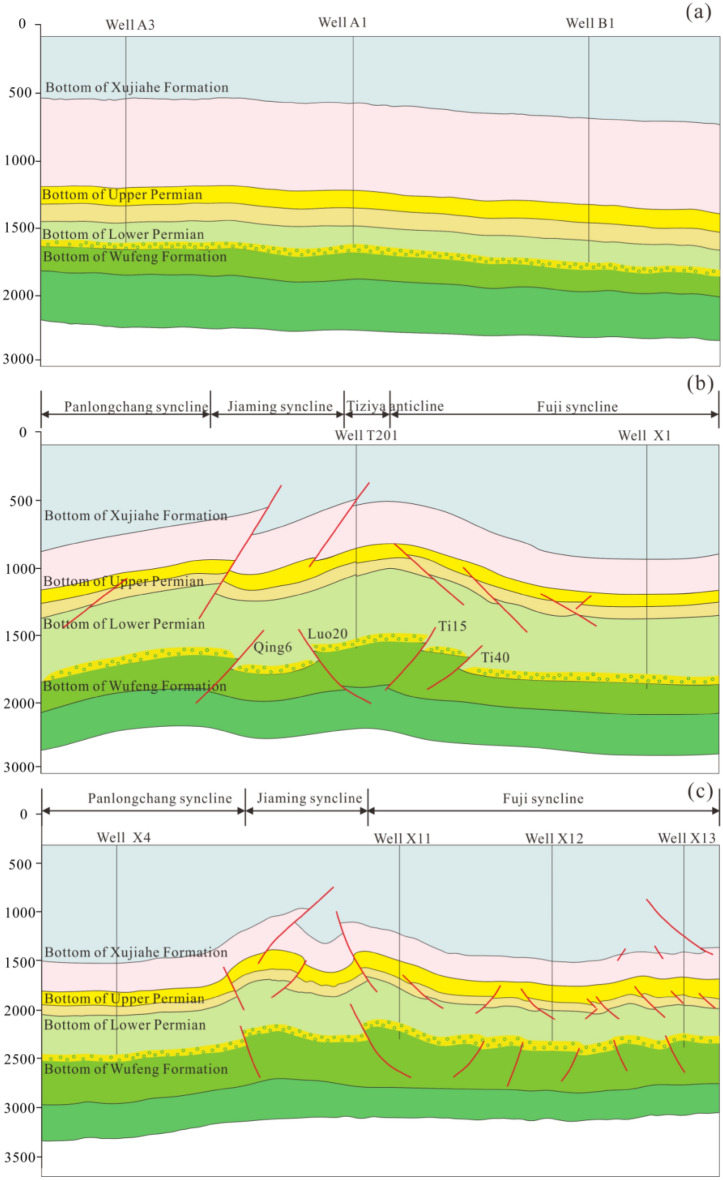
Schematic diagram of the sealing of the roof and floor in the Weiyuan and Luzhou areas. (**a**) Well A3-Well A1-Well B1 in the Weiyuan area, (**b**) Well T201-Well X1 in the Luzhou area, (**c**) Well X4-Well X11-Well X12-Well X13 in the Luzhou area (this figure is generated in CorelDRAW 2020 software, https://www.coreldrawchina.com/).

The complex fault system can directly damage the roof and floor seal integrity of the Wufeng–Longmaxi shale, which is a potential risk factor for shale gas loss. The shale gas layers of the Wufeng–Longmaxi Formation in the Weiyuan and Luzhou areas are typically roof-capped and floor-areaed^36^. The roof comprises siltstone, argillaceous siltstone, and marl of the 2^nd^ member of the Longmaxi Formation, and the floor is nodular limestone of the Pagoda Formation with a tight lithology. The Weiyuan area belongs to the complete roof, floor, and shale layers type; there are few cracks in the Wufeng–Longmaxi Formation. The main reason for the roof and floor seal integrity levels is the long distance from the fault zone, such as Well A3 (Fig. 12a). The Luzhou area belongs to the complete roof and floor shale fracture type(Fig. 12b, c); the cracks are mainly concentrated in the Wufeng–Longmaxi shale layer, and roof and floor cracks are rare^37–40^. The fault system exhibits obvious upper and lower stratification, with typical Y- or anti-Y-shaped structures and positive, locally developed, and flower-shaped structures, which are controlled by the breakthrough of the detachment layer and may have strike-slip properties; the layers that are cut through by the fault include the Cambrian–Longmaxi Formation fault and the Wufeng Formation–Permian fault, without large basement–surface faults developed; multiple high and steep anticlines are developed, with large dip angles on both wings, and faults are developed at the top of the structure; syncline structures are wide and gentle, and the overall structural combination forms typical ejective folds^41–44^.

## Conclusion

1. The organic matter pores in shallow shale reservoirs are in the formation and consolidation period; the pore size gradually increases, and the pore-in-pore phenomenon occurs. There are few organic matter pores with a pore size of 100–200 nm, while the organic matter pores with sizes > 200 nm dominate. The deep shale reservoir is deeply buried, with strong compaction, and it is in the stage of continuously increasing burial depth. The organic matter pores of the shale reservoir in each well are in the growing period; the pores are well preserved and exist mostly as single-round pores. The diameters of organic matter pores are mainly 100–200 nm, and due to strong compaction, microcracks often form due to compaction.
2. The shapes of the hysteresis loop of the pore morphologies of shale reservoirs correspond to the H_2_ type, and all are ink bottle-type pores. The pore volume of shale in each reservoir facies in the Longyi_1_ sub-member ranges from 25 to 30 μL/g, and the pore specific surface area of overmature organic-rich calcareous shale in sublayer 1 is the highest at approximately 48 m^2^/g. The TOC contents of shale reservoirs positively correlate with the porosity, pore volume, and pore specific surface area values. Shale reservoirs mainly develop organic matter pores, and pyrobitumen pores are the main types of organic matter pores. With the increase in the siliceous mineral content of the Longyi_1_ sub-member, the pore volume increases, and the pore specific surface area has no obvious trend. Clay minerals are weakly correlated with the pore volume and positively correlated with the pore specific surface area. Carbonate minerals are not related to the pore volume or specific surface area.
3. The period with the optimally matched controlling factors of the reservoir evolution process, such as the hydrocarbon production, storage capacity, direct caprock, and indirect caprock, in the Longyi1 sub-member is key for shale gas enrichment. The key period of shale gas enrichment occurs from the Middle to Late Triassic to the present; thus, the gas-bearing property is good, and the highest total gas content of the reservoir is 12 m^3^/t. The vertical formation pressure inhibits the lateral migration of shale gas along the shale bedding, and the large-angle intersection of the highly filled fractures and the crustal stress effectively enhances the sealing of the fault and inhibits the vertical escape of shale gas, forming effective 3D closure conditions.

## Data Availability

The data are available from the corresponding author upon reasonable request.
